# A Mutant of *Vibrio parahaemolyticus* *pir*AB_VP_ (+) That Carries Binary Toxin Genes but Does Not Cause Acute Hepatopancreatic Necrosis Disease

**DOI:** 10.3390/microorganisms8101549

**Published:** 2020-10-08

**Authors:** Luis Fernando Aranguren Caro, Hung N. Mai, Siddhartha Kanrar, Roberto Cruz-Flores, Arun K. Dhar

**Affiliations:** Aquaculture Pathology Laboratory, School of Animal and Comparative Biomedical Sciences, University of Arizona, 1041 E. Lowell Street., Tucson, AZ 85721, USA; lfarangu@email.arizona.edu (L.F.A.C.); hungmai@email.arizona.edu (H.N.M.); kanrars@gmail.com (S.K.); robertocruz@email.arizona.edu (R.C.-F.)

**Keywords:** acute hepatopancreatic necrosis disease, Early Mortality Syndrome, shrimp, diseases

## Abstract

*Vibrio parahaemolyticus* carrying binary toxin genes, *pir*AB, is one of the etiological agents causing acute hepatopancreatic necrosis disease (AHPND) in shrimp. This disease has emerged recently as a major threat to shrimp aquaculture worldwide. During a routine PCR screening of AHPND-causing *V. parahaemolyticus* strains, an isolate tested PCR positive for *pirB* (R13) and another isolate tested positive for both the *pir*A and *pir*B (R14) genes. To evaluate the pathogenicity of these isolates, specific pathogen-free (SPF) *Penaeus vannamei* were experimentally challenged. For both R13 and R14 isolates, the final survival rate was 100% at termination of the challenge, whereas the final survival with the AHPND-causing *V. parahaemolyticus* was 0%. The nucleotide sequence of the plasmid DNA carrying the binary toxin genes revealed that R13 contains a deletion of the entire *pirA* gene whereas R14 contains the entire coding regions of both *pir*A and *pir*B genes. However, R14 possesses an insertion upstream of the *pir*A gene. In R14, mRNA for both *pir*A and *pir*B genes could be detected but no cognate proteins. This shows that the genome of AHPND-causing *V. parahaemolyticus* is highly plastic and, therefore, detection of the *pir*A and *pir*B genes alone by DNA-PCR is insufficient as a diagnostic test for AHPND.

## 1. Introduction

Since 2009, acute hepatopancreatic necrosis disease (AHPND), also known as “Early Mortality Syndrome (EMS)”, has caused significant production losses in many South East (SE) Asian countries, including China, Vietnam, Malaysia, Thailand [1,2] and the Philippines [3]. In 2013, the presence of AHPND was confirmed in the Western Hemisphere, particularly in Mexico [4], and in 2017 in the US [5]. In recent years, there have been some studies that suggest the presence of AHPND in other countries in Central America [6] and South America [7,8]. This emerging disease has caused an estimated loss of USD 15.0 billion in shrimp aquaculture worldwide (D.V. Lightner, pers. communication). Due to its rapid spread, AHPND is now posing a serious threat to shrimp farming globally. 

The causative agents of AHPND were determined to be distinct strains of bacteria, including *Vibrio parahaemolyticus* [2,9], *V campbellii* [10] *V. harveyi* [11], *V. owensii* [12] and *V. punensis* [8], that contain a binary toxin, PirAB*vp*, and the PirA and PirB toxins have been confirmed as the virulence factor of the disease. The *pir*AB*_vp_* genes are located in a large plasmid (69–70 kb) within a 3.5-kb fragment flanked by transposes that could be involved in insertion and deletion events causing mutations in the *pir*AB region that might affect the pathogenicity of AHPND *Vibrio* strains [9,12,13,14]. 

AHPND usually appears during the first 20 to 30 days of culture, causing up to 100% mortality in severe cases; however, some reports indicate that juveniles of *Penaeus vannamei* and *Penaeus monodon* of sizes between 6–15 g can be also affected [3]. In the acute phase, the infected hepatopancreas (HP) shows tubule epithelial degeneration and round up and detachment of tubule epithelial cells from the basal membrane. This leads to the sloughing of epithelial cells into the tubule lumen. In the terminal stage, the hepatopancreas shows extensive inter-tubular, hemocytic aggregations which cause hepatopancreas dysfunction and a severe secondary *Vibrio* spp. infection [1,2]. In recent studies, a new phase of AHPND was described, the chronic phase, characterized by the presence of a granulomatous response in the HP tubules accompanied by bacteria and resembling septic hepatopancreatic necrosis (SHPN) [15].

In this study, we found two novel mutants that tested positive for one or both toxin genes (*pir*A and *pir*B) by the World Organization for Animal Health (OIE)-approved PCR method but did not cause AHPND in experimental bioassays, even when the bacterial isolate (R14) contained both toxin genes. The findings revealed that the genome of *V. parahaemolyticus* that cause AHPND is highly plastic, and this has implications in the disease manifestation and diagnosis of AHPND in shrimp using PCR-based diagnostic tools.

## 2. Materials and Methods 

### 2.1. Wild Type and Natural Mutants of AHPND-Causing V. parahaemolyticus 

Four *V. parahaemolyticus* isolates from the University of Arizona Aquaculture Pathology Laboratory were used in this study. *Vibrio parahaemolyticus* 13-028/A3 (A3), an AHPND-causing strain which was isolated from AHPND-affected shrimp in Vietnam [2], was used as a VP_AHPND_ reference strain. In addition, two *V. parahaemolyticus* isolates, R13 and R14, originating from a Latin American country that tested positive by PCR for *pir*B only (R13) or both *pir*AB (R14), and a human clinical *V. parahaemolyticus* strain obtained from the American Type Culture Collection (ATCC strain) that does not contain the toxin genes *pir*AB were used in the bioassay. The ATCC strain served as a negative control for the assay. Bacterial identifications were carried out using 16S rRNA sequencing [16] and *Vibrio*-specific PCR assays targeting the toxR gene [17]. These bacteria were grown on Tryptic soy broth with 2% NaCl (TSB+) at 28–29 °C in a shaker incubator (at 100 rpm for 24 h) prior to the immersion challenge. Bacterial genomic DNA was extracted using the Qiagen DNeasy Kit and following the manufacturer’s protocol (Hilden, Germany). 

### 2.2. Duplex PCR Assay for the Detection of pirA- and pirB-Genes

The *pir*A and *pir*B genes were detected by duplex PCR using PuReTaq ready-to-go PCR beads. The primers used were VpPirA-284F TGA CTA TTC TCA CGA TTG GAC TG/R VpPirA-284R CAC GAC TAG CGC CAT TGT TA that amplifies a 284 bp amplicon of the *pir*A gene and VpPirB-392F VpPirB-392F TGA TGA AGT GAT GGG TGC TC/R VpPirB-392R TGT AAG CGC CGT TTA ACT CA that amplifies a 392 bp region of the *pir*B gene. Amplifications were performed with the following parameters: an initial denaturation at 94 °C for 3 min, followed by 35 cycles of 94 °C for 30 s, 60 °C for 30 s and 72 °C for 30 s, and a final extension at 72 °C for 7 min [18]. Following PCR, an aliquot of the PCR products was analyzed in a 1.5% gel containing 1 × gel red (Biotium, Fremont, CA, USA), and the gel was photographed using a BIORAD Gel Doc XR+ imaging system (BioRad, Hercules, CA, USA). 

### 2.3. Reverse Transcriptase-PCR for the Detection of the pirAB Genes

Total bacterial RNA was extracted from an overnight culture of each bacterial isolate using the RNAzol kit (Molecular Research Center, Houston, TX, USA) and treated with Ambion DNase I (Thermo Fisher Scientific, Waltham, MA, USA) before carrying out cDNA synthesis using the Superscript IV first strand synthesis system (Thermo Fisher Scientific, Waltham, MA, USA). The cDNA was used in the duplex PCR for the detection of *pir*A and *pir*B-like genes, as described above [18]. 

### 2.4. AHPND Challenge Test

Specific pathogen-free (SPF) *P. vannamei* purchased from a commercial vendor in the US were used for the AHPND challenge tests. The AHPND experimental challenge was conducted to determine the pathogenicity of *V. parahaemolyticus* isolates, R13 and R14. The reference strain VP_AHPND_ (A3) was used as a positive control for the bioassay. The challenge was conducted using eight 3-L tanks and artificial seawater (salinity of 25 ppt at 28 °C). Three SPF *P. vannamei* (average weight 7.1 g) were stocked in each jar and two jars were used for each bacterial isolate. Animals were challenged by immersion with *V. parahaemolyticus* R13, *V. parahaemolyticus* R14, *V. parahaemolyticus* ATCC and VP_AHPND_ following a published protocol [10]. An overnight culture broth was added into the jars to obtain a final concentration of 5.5 × 10^5^ CFU mL^−1^. Initial survival was defined by the total number of shrimp present in the tank prior to the AHPND challenge. Mortality was recorded daily from the start of the experiment and moribund shrimp were fixed in Davidson’s alcohol-formalin-acetic acid (AFA) fixative for histopathological analysis [19]. Samples of the dead shrimp were collected to determine the presence of AHPND by PCR. All survivors were fixed at the end of the challenge for histopathological analysis. The duration of the assay was 6 days. 

### 2.5. Histopathology 

The Davidson’s alcohol-formalin-acetic acid (AFA)-fixed samples were processed, embedded in paraffin and sectioned (4 μm thick) in accordance with standard methods [19]. After staining with hematoxylin and eosin (H&E), the sections were analyzed by light microscopy. Infection severity was scored from Grade 0 to Grade 4 (G0–G4) according to Lightner (1996), with G0 being the absence of the disease and G4 indicating the presence of severe lesions and advanced tissue destruction. In the case of lipid droplets in hepatopancreas, the same grading system was used with G0–G1 being an absence and/or very few lipid droplets, respectively, and G3 and G4 indicate a high content of lipid droplets.

### 2.6. Sequence Analysis

The plasmid DNA sequence containing the binary toxin genes *pir*AB in the AHPND reference strain A3 (GenBank KM067908), *V. parahaemolyticus* R13 (GenBank CP028346) and *V. parahaemolyticus* R14 (GenBank CP028145.1) were taken for sequence analyses. A 5.5 kb region carrying the *pir*AB genes located in the plasmid pVPA3-1, R13 and R14 were analyzed to determine potential transposases (IS finder: http://www-is.biotoul.fr). Putative promoter regions and transcription termination sites were predicted by the Bacterial Promoter Prediction Program (BPROM) (http://www.softberry.com/cgi-bin/programs/gfindb/bprom.pl) and ARNold (http://rna.igmors.u-psud.fr/toolbox/arnold/index.php) web tools, respectively. 

To identify the extent of *pir*AB deletion in R13, we cloned and sequenced the corresponding region from the plasmid pVpR13_71Kb of the R13 strain. The amplification was performed using forward primer, A3-15554-FP (15,554–15,574 position of pVPA3-1 sequence 5’-CTT GTA TCT ACC GCG ATA TGC-3’) and reverse primer, pirBFull-1RP (18,862–18,837 position of pVPA3-1 sequence 5’- CTA CTT TTC TGT ACC AAA TTC ATC GG-3’). The PCR product was cloned in a TOPO^TM^ cloning vector, pCR2.1^TM^. The insert was sequenced in both directions and the sequence was deposited into the NCBI database with the accession number MK368635. To identify the insertion sequence in R14, the forward primer R14-66011F (nucleotide positions 66,011 to 66,028 in pVpR14_74Kb plasmid): 5’-CTT CAA GCG TCC CAT TCG-3’ and reverse primer pirA-240R (nucleotide positions 68,018 to 67,997 in pVpR14_74Kb plasmid): 5’-TTT CAT CAC GTT GTA CCA CAT G-3’ were used to clone a fragment that included upstream of the coding region of the *pir*A gene through 240 bp 5’-end of the coding region of *pir*A gene. The amplicon was cloned in a TOPO pCR2.1 vector and sequenced in both directions and the sequence was submitted to the NCBI (accession number MK368636). 

### 2.7. Western Blot Analysis

To detect binary toxins PirA and PirB that cause AHPND by Western blot analysis, *V. parahaemolyticus* ATCC, A3, R13 and R14 were grown in the TSB+ medium for 24 h. Total protein was extracted from the whole culture following previous publication [20]. The total protein was separated on an kilodalton (kD)precast SDS-PAGE gel (BioRad) using a running buffer (25 mM Tris, pH 8.3, 192 mM glycine, 0.1% SDS) at 80 V for 1 h. The proteins present in the gel were transferred onto a nitrocellulose membrane at 1mA/cm^2^. Western blot analysis was conducted following a published protocol [20]. Anti-PirA and -PirB monoclonal (GenScript Biotech) antibodies at a concentration of 1:5000 were used. In addition, an anti- Glyceraldehyde 3-phosphate dehydrogenase (GAPDH) monoclonal antibody (Abcam®, UK) was used to detect GAPDH, a loading control, the molecular mass of which varies between 35 and 37 kDa depending on bacterial isolates. 

## 3. Results

### 3.1. Duplex PCR for the Detection of the pirA and pirB-Like Genes

**Figure 1:**
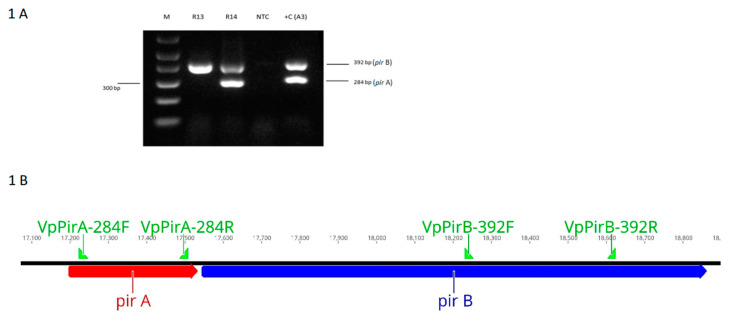
(**A**). A photograph of *pir*A and *pir*B amplicons generated by DNA PCR from *Vibrio parahaemolyticus* isolates carrying either one or both of the toxin genes. M = 1 kb molecular marker; R13 = *V. parahaemolyticus* R13 strain; R14 = *V. parahaemolyticus* R14 strain, *V. parahaemolyticus* ATCC strain; NTC = non-template control, +C (A3) = reference strain of *V. parahaemolyticus* causing acute hepatopancreatic necrosis disease (AHPND). (**B**). A description of the toxin genes *pir*AB showing Forward and Reverse primer binding sites.

Figure 1 shows the amplification of the *pir*A and *pir*B genes by DNA PCR using total genomic DNA of *V. parahaemolyticus* isolates from Latin America. In the *V. parahaemolyticus* reference strain (A3), amplicons of 284 bp (*pir*A) and 392 bp (*pir*B) were detected. In the isolate R13, only the amplicon corresponding to *pir*B (392 bp) was detected, whereas in R14, both *pir*A and *pir*B genes were successfully detected. 

### 3.2. Challenge Test, Histopathology and PCR Results 

**Figure 2:**
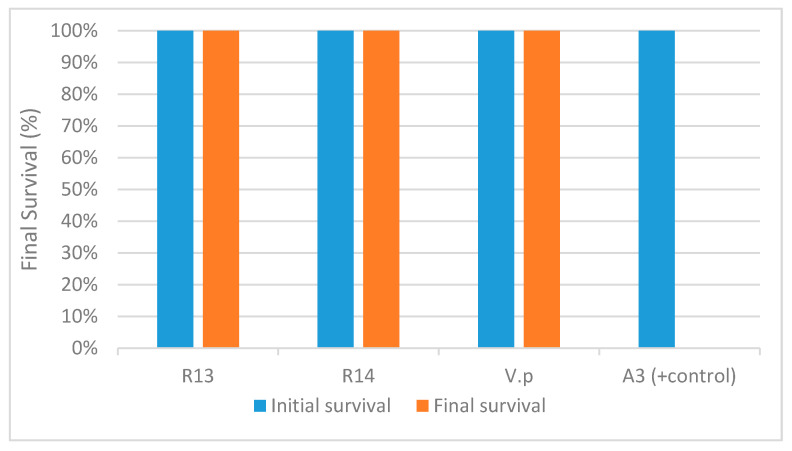
Final survival rate of shrimp, *Penaeus vannamei*, experimentally infected with four different isolates of *Vibrio parahaemolyticus*—R13, R14, A3 (reference AHPND strain) and VP ATCC strains. Data set represents the mean of two replicates for each strain.

**Figure 3:**
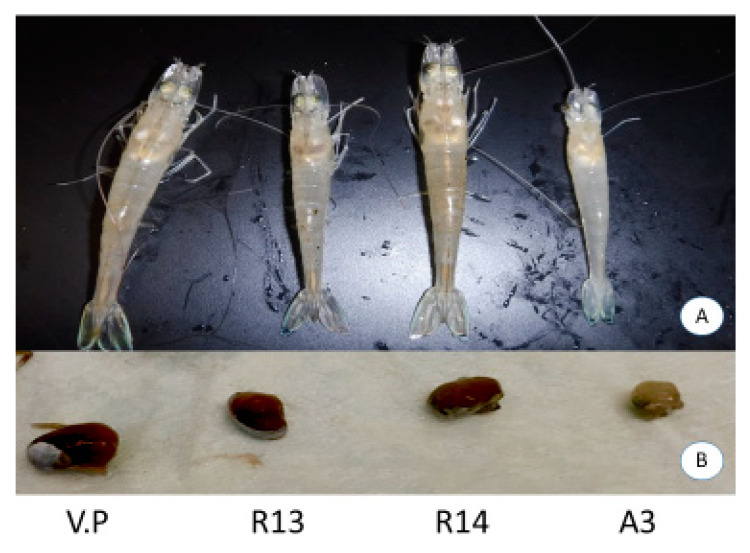
Clinical signs of shrimp, *Penaeus vannamei,* experimentally infected with four different isolates of *Vibrio parahaemolyticus*—*V. parahaemolyticus* ATCC strain (V.P), R13, R14 and A3 (AHPND reference strain). Panel (**A**): Shrimp on the left shows a normal color in the hepatopancreas and the shrimp on the right shows a pale discoloration on the hepatopancreas and an empty stomach—typical signs of AHPND-infected shrimp. The control shrimp (unchallenged) and the shrimp challenged with R13 and R14 all showed a normal color (dark brown) of hepatopancreas while all the shrimp challenged with the reference strain A3 showed a pale discoloration of the hepatopancreas. Panel (**B**) shows a sample of a typical hepatopancreas dissected from shrimp exposed to R13, R14 and A3. The shrimp infected with the A3 strain shows an atrophied and pale hepatopancreas.

The experimental challenge using the *V. parahaemolyticus* reference strain A3 resulted in 100% mortality of the SPF shrimp at 1 day post-challenge. However, no mortalities were recorded in animals challenged with either R13, R14 or *V. parahaemolyticus* ATCC strains (Figure 2). Dorsoventral sectioning of the cephalothorax of healthy and bacterial challenged shrimp is shown in Figure 3. Hepatopancreas samples from R13, R14 and A3 showed different discoloration patterns. Shrimp infected with the A3 strain showed clinical signs characteristics of AHPND, including pale discoloration and atrophy of hepatopancreas (Figure 3b). In contrast, animals challenged with R13, R14 and ATCC *V. parahaemolyticus* strains did not show any clinical signs and hepatopancreas did not display any discoloration or atrophy in the challenged animals. In deceased animals challenged with A3, both *pir*A and *pir*B genes could be amplified by duplex PCR. In R14, both *pir*A and *pir*B genes could be amplified by duplex PCR from challenged shrimp. In the R13 strain, only *pir*B and not the *pir*A gene could be amplified, while in *V. parahaemolyticus* ATCC strain, neither of the toxin genes could be amplified. 

**Figure 4:**
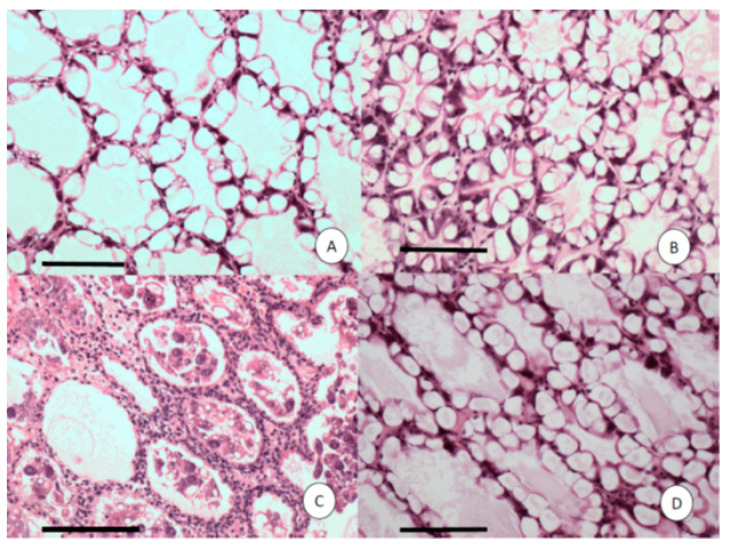
Histological sections of the hepatopancreas (HP) of *Penaeus vannamei* shrimp challenged with four strains of *Vibrio parahaemolyticus*. (**A**) V.P-ATCC; (**B**) R13; (**C**) VP_AHPND_ strain A3; (**D**) R14 isolate. Only C displays acute HP necrosis with severe sloughing of HP tubule epithelial cells. Mayer–Bennett H&E. Scale bars = 100 μm.

Histopathology of shrimp challenged with VP_AHPND_ A3 showed lesions typical of AHPND in the acute phase, including multifocal necrosis and massive sloughing of hepatopancreatic tubule epithelial cells in the medial region of the hepatopancreas and progressing outward to the distal region (Figure 4C). However, in the shrimp exposed to R13, R14 or VP, no lesions that are characteristic of AHPND were observed. High levels of vacuolization (G3) in the hepatopancreatic tubule were observed in all the examined shrimp. Although there are no apparent lesions in the shrimp challenged with the R13 and R14 strains, the hepatopancreatic tubules in the shrimp challenged with R13 look smaller than those in the shrimp challenged with the R14 strain. This could have been due to the sectioning of the HP tubule in a region closest to the apical end on the shrimp exposed to the R13 strain that tend to be smaller than other sections close to the proximal end.

### 3.3. Detection of pirA and pirB Transcripts by RT-PCR 

**Figure 5:**
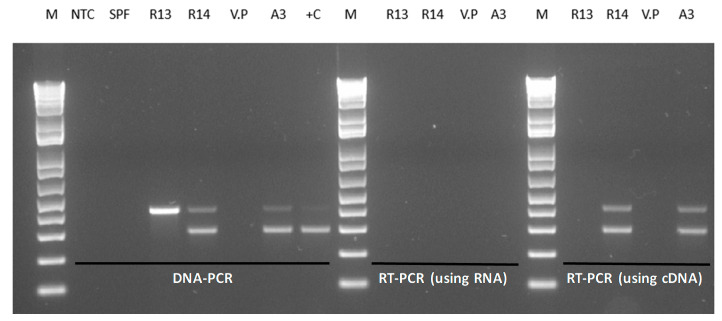
Polymerase chain reaction amplification of *pir*AB genes. Amplification of *pir*AB genes was performed using total genomic DNA (DNA-PCR), total RNA without synthesizing cDNA (using RNA) or after synthesis of cDNA (using cDNA). M = 1 kb molecular ladder, NTC = no template control in PCR, SPF = specific pathogen-free *Penaeus vannamei,* R13 = *V. parahaemolyticus* R13 strain, R14 = *V. parahaemolyticus* R14 strain, V.P = *V. parahaemolyticus* ATCC strain, A3 = reference AHPND strain, +C = positive control.

The binary toxin genes *pir*A and *pir*B were detected in shrimp challenged with R14 strains, while only the *pir*B gene was detected in the shrimp challenged with R13 (Figure 1). To further confirm that the toxin genes are not expressed, total RNA was extracted and cDNA was used to perform RT-PCR to amplify the *pir*A and *pir*B genes. A photomicrograph of an agarose gel showing cDNA amplicons representing the binary toxin genes amplified by duplex RT-PCR is presented in Figure 5. The *pir*A and *pir*B genes could not be amplified by RT-PCR in R13; meanwhile, R14 and A3 strains displayed expression of the toxin genes (Figure 5). 

### 3.4. Sequence Analyses

**Figure 6:**
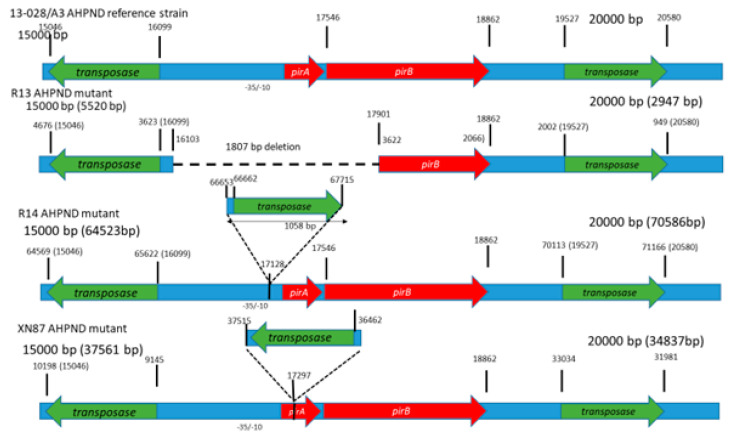
Comparison of the normal pVA3 plasmid (strain A3) and their mutated forms in isolate R13 and R14. (a) Scaled diagram of the *pir*A and *pir*B toxin gene regions in the normal pVA3 plasmid. (b) Mutant strain R13. Dash lines denote the absence of *pir*A. (c) Mutant strain R14. Notice insertion upstream of *pir*A. (d) XN87 AHPND mutant. The numberx over the gene organization schematics indicate the nucleotide position.

**Figure 7:**
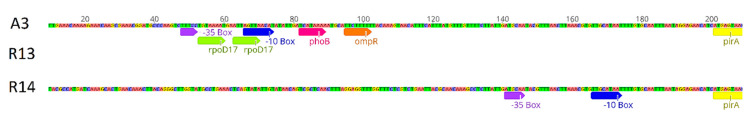
Promoter regions of A3, R13 and R14 and transcriptional factor binding sites; the descriptions of each site and their location are shown. Using BPROM in *V. parahaemolyticus* strain A3, the transcriptional factors, −35 box, rpoD17 (transcriptional binding site), −10 box, phoB (transcriptional regulatory protein PhoB site) and ompR (transcriptional regulatory protein OmpR site) were identified. In strain R13, this entire genomic region is deleted, and in the R14 strain, −35 box and −10 box were identified.

The genetic features of the plasmid DNA carrying the binary toxin genes in the *V. parahaemolyticus* reference strain A3 and the mutant strains R13 and R14 are summarized in Table 1 and Figure 6. In the putative promoter region upstream of the *pir*AB genes in the R13 strain, there is a deletion which included the entire predicted promoter region. In contrast, the R14 strain contains the computationally predicted promoter region containing a -10 (AGTTAACAT) and a -35 (TTTCCT) at the nucleotide positions 66,586 and 66,568 bp, respectively, with reference to the GenBank entry CP28145.1 [21]. Both strains R13 and R14 contain a putative transcription termination site potentially forming a stem-loop secondary structure (stem sequence, AACCGTCC, and the loop being, ATTA) (Figure 7).

Using the same set of primers used for the DNA-PCR assay, the RT-PCR assay successfully amplified both *pir*A and *pir*B genes using total RNA from overnight cultures of R14 but not the R13 strain, indicating that the binary toxin genes are transcribed in R14 but not in R13. Duplex RT-PCR also detected both *pir*A and *pir*B genes in the reference strain A3. 

The R13 strain has a deletion at nucleotide positions 16,103 to 17,910, corresponding to the pVPA3-1 plasmid. The deletion represented the promoter sequence upstream of *pir*A, the complete *pir*A coding-sequence and the 5’-portion of *pir*B coding sequence (nucleotide position 1–364 at the 5’-end of the *pir*B gene). Because of this deletion, the *pir*AB genes are not transcribed, and consequently, no cDNA product was detected (Figure 5).

In the R14 strain, a 1058 bp insertion was observed 59 bp upstream of the start codon of the *pir*A gene. This represented an insertion sequence 5 (IS5) class of sequence elements [22]. It codes for a transposase enzyme that is required for DNA transposition. The A3 strain plasmid (pVPA3-1) carries two of these IS5 elements, one at nucleotide position 15,046 to 16,099 (right to left orientation of the element based on transposase gene position orientation) and a second one at position 19,527 to 20,581 (left to right orientation of the element). The R14 strain carries three copies of the IS5 element: one copy at the position of 64,568 to 65,622 (right to left orientation), a second one at the position of 70,113 to 71,169 (left to right orientation), and the third copy at position 66,662 to 67,718 (left to right orientation) (Figure 7). The third position insertion is surrounded by a 5 bp direct repeat of ATTGA at both ends of the insertion. Such direct repeats of small sequences at both ends (here, a 5 bp fragment) are a landmark of the IS element insertion. This insertion is located at −58 compared to the transcription initiation codon of the *pir*AB gene block. This insertion disrupts the normal position of transcription elements of native pVPa3-1 plasmid due to addition of an extra 1054 bp IS5 element in pVpR14_74Kb plasmid of R14. However, the insertion did not disrupt the transcription since both *pir*A and *pir*B were detected by RT-PCR (Figure 5).

The promoter region (634 bp) of A3 and R14 was compared with BPROM software of softberry suite. The A3 promoter contains phoB, carP, argR and ompR transcription factors’ binding sites which are absent in case of the R14 promoter. The phoB transcription factor is involved in *Vibrio cholerae* virulence and phosphate starvation [23]. The ompR regulates osmotic stress in *Escherichia coli* via outer membrane porins [24] (Figure 7).

### 3.5. Detection of Binary Toxin by Western Blot Analysis

**Figure 8:**
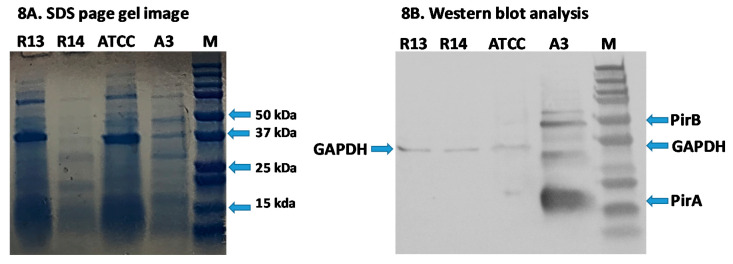
SDS-Page and Western blot analyses of proteins extracted from *Vibrio parahaemolyticus* R13, R14, ATCC and A3 strains. (**A**) SDS-Page gel image. (**B**) Western blot analysis. For each bacterial isolate, three micrograms of total protein were loaded into each well. Monoclonal antibodies against GAPDH (loading control), PirA, and PirB were used to detect corresponding proteins. Lane 1, *V. parahaemolyticus* R13 strain; lane 2, V. parahaemolyticus R14 strain; lane 3 *V. parahaemolyticus* ATCC strain; lane 4, *V. parahaemolyticus* A3 strain; lane M, 250kD protein ladder. The location of PirB (~50 kDa), GADPH (~37 and ~ 35 kDa) and PirA (~17 kDa) proteins are indicated in the Western blot analysis image.

Using PirA and PirB polyclonal antibodies, both PirA and PirB toxins were detected in the reference strain A3 and were not detected in the R13, ATCC and R14 strains. (Figure 8a,b). The results confirmed that the binary toxin is only expressed in the *V. parahaemolyticus* reference strain A3. The toxins are not expressed in R13 and R14 strains, despite the fact that both *pir*A and *pir*B transcripts were detected in R14 by RT-PCR. 

## 4. Discussion

In this study, we found two mutant non-pathogenic strains of *V. parahaemolyticus*, R13 and R14, that harbor the pVA3 plasmid and contain either *pir*A or both *pir*AB genes but did not cause mortality in shrimp in a laboratory experimental challenge. These findings have important implications for AHPND diagnosis since detection of the *pir*A and *pir*B genes alone is insufficient to identify AHPND-causing *Vibrio* spp. Using RT-PCR, it was determined that the R13 strain did not produce *pir*AB mRNA while R14 did produce mRNA for both *pir*A and *pir*B genes, although no toxin protein was detected by Western blot analyses. Han and colleagues [10] proposed a classification scheme to categorize VP_AHPND_ strains based on the presence or absence of the *pir*A and *pir*B genes. Bacteria that carry both toxin genes and cause AHPND are categorized as Type I, while those that carry either of the toxin genes and do not cause AHPND are categorized as Type II. Based on this classification scheme, the R13 mutant fits into the Type II category. However, the R14 strain does not meet the criteria for either Type I or Type II mutants since it contains both *pir*A and *pir*B genes without causing AHPND. Therefore, in order to accommodate a unique mutant, such as the R14 strain, we propose, here, a new group of mutants—Type III. This class of mutant contains the entire coding regions of both *pir*A and *pir*B genes and contains insertion(s) within or just outside the Open reading frame (ORF) of these genes that make the corresponding strain non-pathogenic. Interestingly, the insertion detected in the R14 strain is unique in that it did not affect the transcription of the cognate mRNA but inhibited translation of PirAB proteins. Hence, this strain did not cause mortality in *P. vannamei* shrimp in the experimental challenge. Only the *V. parahaemolyticus* reference strain (A3) produced PirA and PirB toxins and caused AHPND in the experimental challenge. It was reported that in order to cause AHPND, both components of the binary toxin *pir*AB are needed. Sirikharin et al. [13] were able to produce AHPND with 100% mortality only when both recombinant PirA and PirB proteins were injected into *P. vannamei* shrimp at 10 µg/g of shrimp. However, when each of these proteins was injected separately, AHPND could not be reproduced in shrimp. We conclude that in R13, the deletions of the *pir*A gene disrupt the expression of the corresponding toxin and, therefore, this strain cannot produce the binary toxin needed to cause AHPND. However, the strain R14 is unique in being able to express the *pir*AB genes, yet it does not produce a functional toxin that cause lesions characteristic of AHPND in shrimp. It is known that translation in bacteria can be disrupted due to several factors. For example, in *Escherichia coli* and *Bacillus subtilis*, mRNA can be destabilized by mutations in the transcript which interfere ribosome binding resulting in translational disruption [25,26,27]. In *E. coli*, the translation can also be disrupted by uncoupling transcription and translation in which the coding region is exposed to RNase. Typically, ribosome translates mRNA at a speed which is similar to the speed of transcription [28]. Therefore, the nascent transcript carry several ribosomes closely behind the T7-RNA polymerase [29]. Occasionally, T7-RNA polymerase synthesizes mRNA at a speed which is several fold of ribosome speed, thereby uncovering mRNA which is exposed to RNase and cleaved by RNase, resulting in a pause in translation [30]. Alternatively, the translation can be incomplete if ribosome is stalled due to termination of transcription before terminal codon [31,32], existence of a rare codon [33], amino acid starvation [34,35] and the presence of the sequence resulting in the elongation factor being arrested [36]. We do not know if any of these mechanisms are functional in R14 causing translational inhibition. 

Insertion sequences (IS) are among the simplest mobile genetic elements. They can move within a genome (within a single DNA molecule or between different DNA molecules) or horizontally between genomes via phages and plasmids. IS-mediated gene regulation affects virulence, microbial resistance and metabolism of the host genome. IS-mediated inactivation of the host gene occurs when it is inserted in a coding sequence of the gene or in the promoter region of the corresponding gene [37]. For example, in *Staphylococcus aureus,* IS256-mediated modulation of virulence has been reported by insertion into rot (repressor of toxins) promoter [38]. Recently, Phiwsaiya et al. [14] isolated a *V. parahaemolyticus* isolate, XN87, that tested negative by PCR for *pir*A gene but carried *pir*B. Sequence analysis revealed that the plasmid carrying toxin genes in XN87 contained an out-of-frame insertion of a transposon gene in *pir*A that caused a 2-base reading frameshift in the remainder of the *pir*A and in the downstream *pir*B gene sequence (Figure 6). RT-PCR assay showed that bicistronic *pir*AB mRNA is not produced, and in Western blot analysis, neither toxins were detected. However, in experimental immersion challenge, a 47% mortality rate was recorded in *P. vannamei* shrimp without any pathognomonic lesions characteristic of AHPND. 

## 5. Conclusions

Some *V. parahaemolyticus* isolates carry mutant pVA plasmids that produce no PirAB toxins and cause no mortality in experimental challenges using SPF shrimp. We do not know the molecular mechanism of translational inhibition in R14 strain, and future studies will focus on elucidating the yet unknown mechanism of translational inhibition. The phoB and ompR transcription factors binding to the A3 promoter may increase virulence and preferential osmotic survival in A3 compared to R14 where these transcription factors are absent. Despite the unknown reason for the lack of binary toxin production in R14, the current finding highlights the fact that PCR-based detection alone cannot identify AHPND causing *V. parahaemolyticus* strains unequivocally, and PCR-based diagnostics of AHPND-positive cases need to be confirmed by a bioassay, histopathology and detection of functional binary toxins, a true predictor of AHPND.

## Figures and Tables

**Table 1:** Genetic features of the plasmid DNA carrying binary toxin gene in the *Vibrio parahaemolyticus* reference strain, A3, and the mutant strains R13 and R14. A recently described mutant strain of *V. parahaemolyticus,* XN87, that carries one of the two toxin genes but causes mortality in *Penaeus vannamei* shrimp is included for comparison [14].

Genetic Features	R13	R14	V.P (ATCC)	A3	XN87 *
Size (kb)	71	75.713	−	69	−
*pir*A	−	+	−	+	−
*pir*B	Truncated (nt 361–1317)	+	−	+	+
Deletion/Insertion	Deletion	Insertion	−	−	Insertion
Mortality (%)	0%	0%	0%	100%	47%

*: Taken from Phiwsaiya et al., 2017 [14].
